# Neoadjuvant chemotherapy in advanced epithelial ovarian cancer by histology: A SEER based survival analysis

**DOI:** 10.1097/MD.0000000000032774

**Published:** 2023-01-27

**Authors:** Yuexi Liu, Meng Ni, Fanfan Huang, Qiuying Gu, Yao Xiao, Xinyue Du

**Affiliations:** a Department of Obstetrics and Gynecology, The first Affiliated Hospital of Chongqing Medical University, Chongqing, China; b International Peace Maternity and Child Health Hospital, School of Medicine, Shanghai Jiao Tong University, Shanghai, China; c Shanghai Key Laboratory of Embryo Original Disease, Shanghai, China; d Department of Ophthalmology, The first Affiliated Hospital of Chongqing Medical University, Chongqing, China; e Department of Cardiovascular medicine, The first Affiliated Hospital of Chongqing Medical University, Chongqing, China.

**Keywords:** advanced ovarian cancer, cause-specific survival, neoadjuvant chemotherapy, overall survival, SEER

## Abstract

To evaluate the prognostic effect of neoadjuvant chemotherapy (NACT) in advanced epithelial ovarian cancer (EOC) patients with different histological subtype. Stage III/IV EOC patients diagnosed between 2010 and 2018 were identified from the surveillance, epidemiology, and end results database (SEER) database and stratified by histological subtype. Kaplan–Meier analysis was used for the assessment of overall survival (OS) cause-specific survival (CSS) before and after matching for baseline characteristics between NACT and primary debulking surgery (PDS) groups. Cox proportional risk model was conducted to identify independent prognostic factors. A total of 13,582 patients were included in the analysis. Of them, 9505 (74.50%) received PDS and 3253 (25.50%) received NACT. Overall, an inferior OS and CSS was observed among patients with high-grade serous carcinoma (HGSC) receiving NACT, while NACT served as a protective factor in clear cell carcinoma and carcinosarcoma in both original cohorts and adjusted cohorts. For other histo-subtypes, PDS showed survival benefit over NACT in certain cohorts of models. Prognostic effect of NACT in advanced EOC differed from pathological subtypes. Although it served as a risk factor for HGSC, patients with less common subtypes may benefit from NACT.

## 1. Introduction

Epithelial ovarian cancer (EOC) is a highly aggressive malignancy, with nearly 70% of patients diagnosed in advanced stages.^[1–3]^ The standard therapy involves primary debulking surgery (PDS) and adjuvant chemotherapy.^[4]^ For advanced patients with extensive tumor dissemination and low possibility of achieving complete cytoreduction, interval debulking surgery (IDS) after neoadjuvant chemotherapy (NACT) has increasingly been offered as a valid alternative.^[5,6]^ Although several European/Asian randomized controlled trials demonstrated non-inferior prognosis of NACT against PDS,^[5,7–9]^ controversy remains about its efficacy, mainly due to several U.S. large-scale observational studies suggesting worse outcomes.^[10–16]^ Given the mixed results of prognosis, researches are ongoing to identify survival-related factors of NACT, which mainly focus on clinicopathological factors such as age, tumor burden, co-morbidities and outcome of debulking surgery,^[10,14,17,18]^ without considering the intrinsic heterogeneity in biology.

Although high-grade serous carcinoma (HGSC) occupies the majority of advanced EOC, it is important to recognize that the spectrum of EOC incorporates a group of heterogeneous tumors, including clear cell, carcinosarcoma, endometrioid, etc.^[19,20]^ Each of the them is associated with distinct clinical and pathological features, resulting in different therapeutic responsiveness.^[21,22]^ However, much of the clinical trials in NACT settings focused on HGSC and didn’t distinguish other subtypes due to limited sample size.^[5,7–18]^ A more detailed evaluation of primary management on different histological subtypes could be helpful to guide the therapeutic decisions. Therefore, we analyzed real-world data from the Surveillance, Epidemiology, and End Results (SEER) Database to explore the prognostic role of NACT-IDS versus PDS for advanced EOC based on pathological subtype.

## 2. Methods

### 2.1. Data source and study population

**Figure 1.**
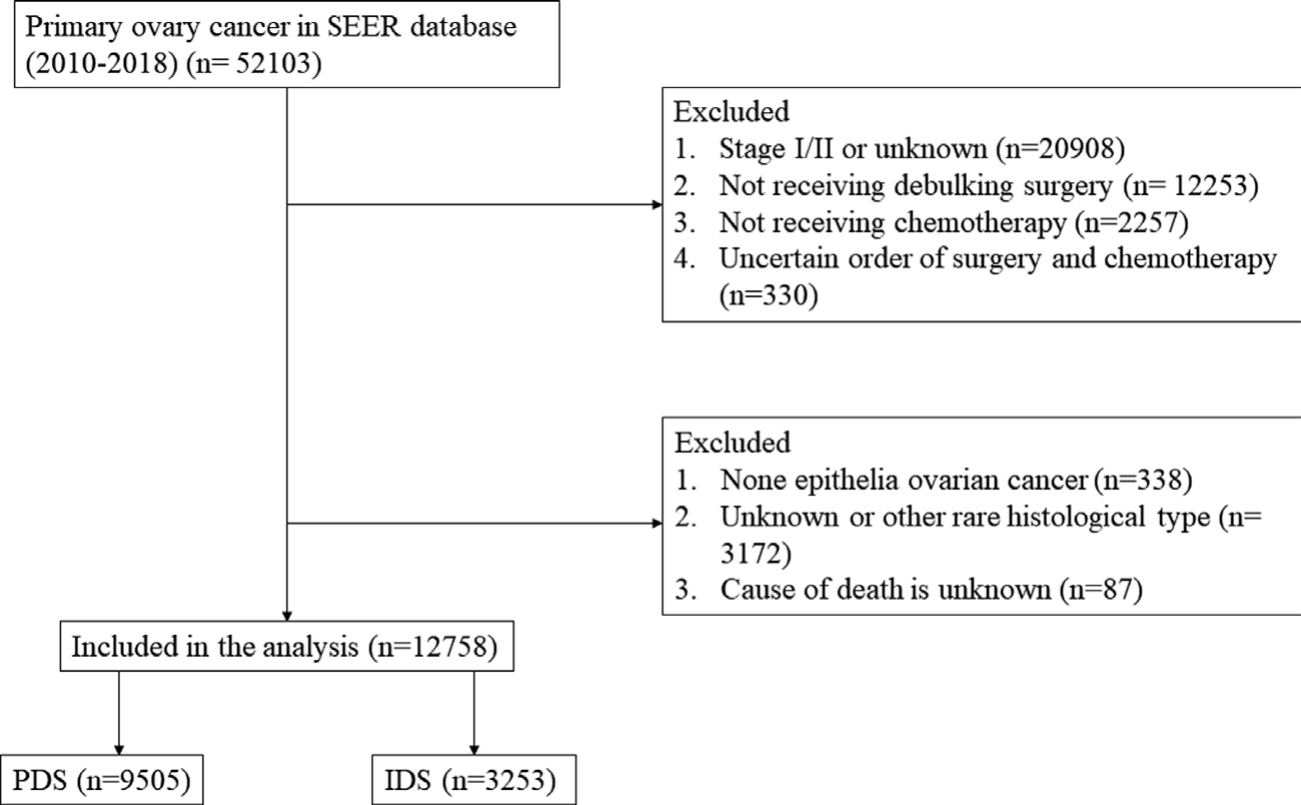
Flowchart of the analysis.

Data of patients diagnosed as primary ovary cancer from 2010 to 2018 were identified and extracted from the SEER database (n = 52,103). The follows patients were excluded: stage 1/2 or unknown (n = 20,908); not receiving debulking surgery (n = 12,253); not receiving chemotherapy (n = 2257) or with uncertain order of surgery and chemotherapy (n = 330); none epithelia ovarian cancer (n = 338); unknown or other rare histological type (n = 3172); and cause of death is unknown (n = 87). Ultimately, 12,758 patients were included, with 9505 confirmed as PDS and 3253 as IDS (Fig. 1). The *International Classification of Diseases for Oncology, Third Edition* were used to identify the following subtypes: HGSC, clear cell carcinoma, carcinosarcoma, endometrioid carcinoma, mucinous carcinoma, low-grade serous carcinoma (LGSC), and mixed cell carcinoma.

### 2.2. Covariates

Demographics included patient age, year of diagnosis, race/ethnicity (white, black, others, and unknown), marital status (married, not married, and unknown). Staging information for each patient was derived from the American Joint Cancer Committee and determined according to the International Federation of Gynecologists and Obstetricians: IIIA, IIIB, IIIC, IIINOS and IV. Other tumor characteristics included tumor grade (I, II, III, IV), laterality (unilateral versus bilateral), volume (≤ 10 cm vs >10 cm), distant metastasis to brain, lung, bone or liver (yes vs no/unknown), pretreatment CA125 (normal/negative, elevated/positive, and unknown). NACT, the exposure variable, was designated according to the sequence of surgery/systemic treatment (PDS vs NACT-IDS). Treatment types included performance of debulking surgery (R0: complete resection, non R0: residual tumor nodules, and unknown), radiation (yes vs no/unknown). Overall survival (OS) was calculated from the date of diagnosis to the date of death due to any cause, censoring, or last follow-up. Cause-specific survival (CSS) was calculated from the date of diagnosis to the date of death due to ovarian cancer.

### 2.3. Statistical analysis

Categorical variables were compared using Pearson chi-square test or Fisher’s exact test. Continuous variables were evaluated with Student’s *t* test or Mann–Whitney *U* test. Propensity score model (PSM) and inverse probability of treatment weight (IPTW) model were constructed to balance the baseline clinicopathological factors. PSM was constructed via a multivariable logistic regression model which included variables significantly associated with treatment modality via univariable analysis and the ones with significant importance in clinical. To construct the PSM of CSS and OS, patients treated with IDS were matched 1:1 to patients treated by PDS on propensity score by using an optimal method. On the basis of the propensity score, an IPTW was calculated and truncated at the 1th and 99th percentiles. OS and CSS were analyzed by the Kaplan–Meier estimates and compared by the log-rank test. Cox proportional hazards model was used to determine independent prognostic factors. All calculations were performed with R 4.0.6.

## 3. Results

### 3.1. Characteristics of the use of NACT among years

Between 2010 and 2018, we identified 13582 patients with primary ovary cancer who were histologically confirmed as described above. A total of 9505 (74.50%) received PDS and 3253 (25.50%) received IDS (see Fig. S1, http://links.lww.com/MD/I386, Supplemental Digital Content). The number of patients receiving NACT plus surgery was at a rising trend (*p* - trend < 0.001).

### 3.2. High-grade serous carcinoma

**Figure 2.**
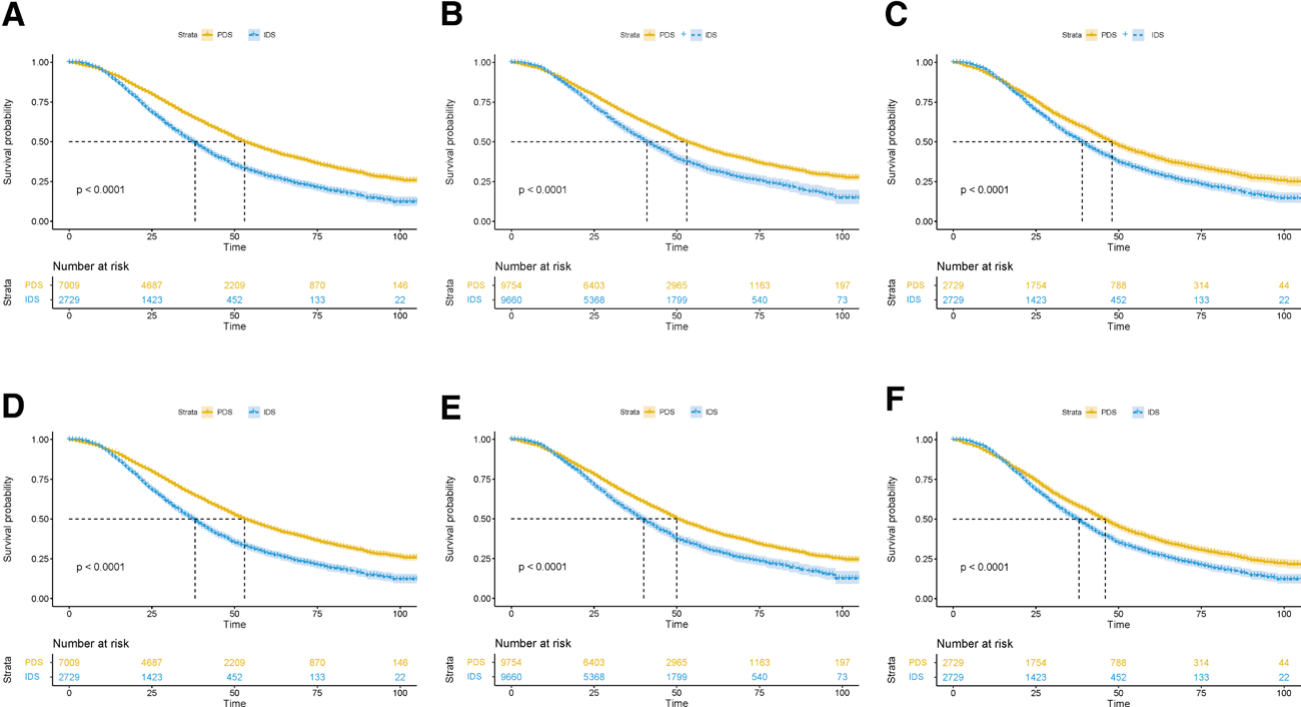
Kaplan–Meier survival curves of CSS and OS in unbalanced (A, D), IPTW-adjusted (B, E), and PSM (C, F) cohorts of patients receiving NACT or PDS in HGSC. CSS = cause-specific survival, HGSC = high-grade serous carcinoma, IPTW = inverse probability of treatment weight, NACT = neoadjuvant chemotherapy, OS = overall survival, PDS = primary debulking surgery, PSM = propensity score model.

Baseline characteristics of the original, IPTW and PSM population with HGSC are shown in Table S1, http://links.lww.com/MD/I391, Supplemental Digital Content. Originally, patients who received IDS were older, with more frequently elevated pretreatment CA125, distant metastasis and International Federation of Gynecologists and Obstetricians stage IV disease. Characteristics were balanced between 2 groups after IPTW and PSM. PDS showed a significant survival benefit over IDS in the unbalanced cohort (OS 53 vs 38 months, *P* < .001; CSS 57 vs 39 months, *P* < .001), IPTW cohort (OS 50 vs 40 months, *P* < .001; CSS 53 vs 41 months, *P* < .001) and PSM cohort (OS 46 vs 38 months, *P* < .001; CSS 48 vs 39 months, *P* < .001, Fig. 2). Multivariate analysis identified IDS as a risk factor for OS in unbalanced cohort (hazard ratio [HR] 1.30; 95% confidence interval [95% CI] 1.22–1.39; *P* < .001), IPTW cohort (HR 1.34; 95% CI 1.25–1.44; *P* < .001) and PSM cohort (HR 1.26; 95% CI 1.17–1.36; *P* < .001). The same results were observed for CSS in unbalanced cohort (HR 1.33; 95% CI 1.24–1.42; *P* < .001), IPTW cohort (HR 1.37; 95% CI 1.27–1.47; *P* < .001) and PSM cohort (HR 1.29; 95% CI 1.20–1.39; *P* < .001, Table S2, http://links.lww.com/MD/I392, Supplemental Digital Content). For other adjusted covariates, age, marriage status, tumor laterality, advanced stage, tumor volume, and residual disease were independent prognostic factors associated with OS in unbalanced cohort and both adjusted cohorts. Likewise, prognostic factors for OS remained statistically significant for CSS.

### 3.3. Clear cell carcinoma

**Figure 3.**
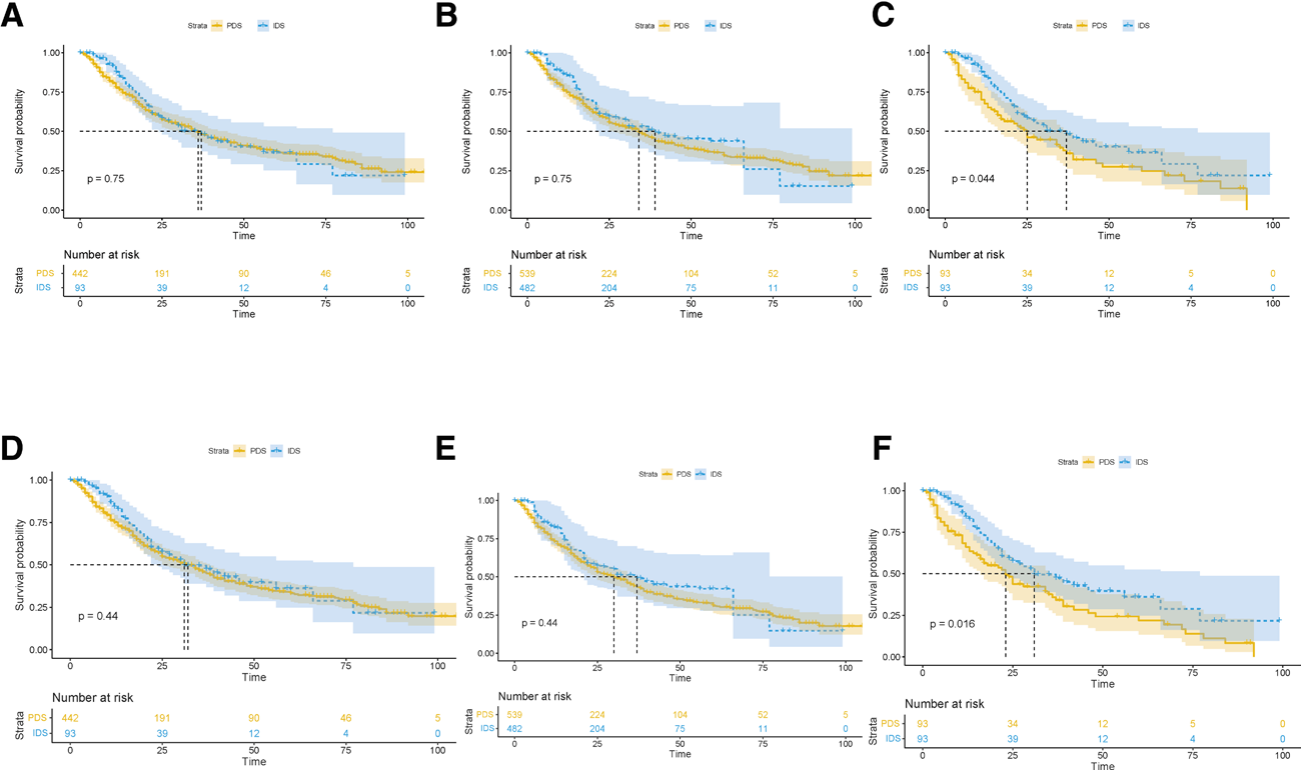
Kaplan–Meier survival curves of CSS and OS in unbalanced (A, D), IPTW-adjusted (B, E), and PSM (C, F) cohorts of patients receiving NACT or PDS in clear cell carcinoma. CSS = cause-specific survival, IPTW = inverse probability of treatment weight, NACT = neoadjuvant chemotherapy, OS = overall survival, PDS = primary debulking surgery, PSM = propensity score model.

Baseline characteristics of the original IPTW and PSM population with clear cell carcinoma are shown in Table S3, http://links.lww.com/MD/I393, Supplemental Digital Content. The original group patients who received IDS were older, with higher rates of bilateral disease, stage IV tumor and elevated pretreatment CA125. Based on the propensity score, treatment groups were comparable in IPTW and PSM cohorts. The PDS group showed no survival benefit over IDS group in unbalanced cohort (OS 32 vs 31 months, *P* = .44; CSS 36 vs 37 months, *P* = .75) and IPTW cohort (OS 30 vs 37 months, *P* = .44; CSS 34 vs 39 months, *P* = .75). However, PSM cohort revealed a better survival of patients receiving NACT-IDS (OS 20 vs 31 months, *P* = .016; CSS 20 vs 37 months, *P* = .044, Fig. 3). After adjusted for prognostic factors, IDS served as a protective factor for OS in unbalanced cohort (HR 0.57; 95% CI 0.40–0.82; *P* = .002) and PSM cohort (HR 0.50; 95% CI 0.33–0.76; *P* = .001, Table S4, http://links.lww.com/MD/I394, Supplemental Digital Content). The same trend was observed in CSS cohort (unbalanced cohort, HR 0.61; 95% CI 0.42–0.88; *P* = .009; PSM cohort, HR 0.52; 95% CI 0.34–0.79; *P* = .002), while not in IPTW cohort. In addition, tumor laterality, marriage status and residual disease were independent prognostic factor for OS and CSS.

### 3.4. Carcinosarcoma

**Figure 4.**
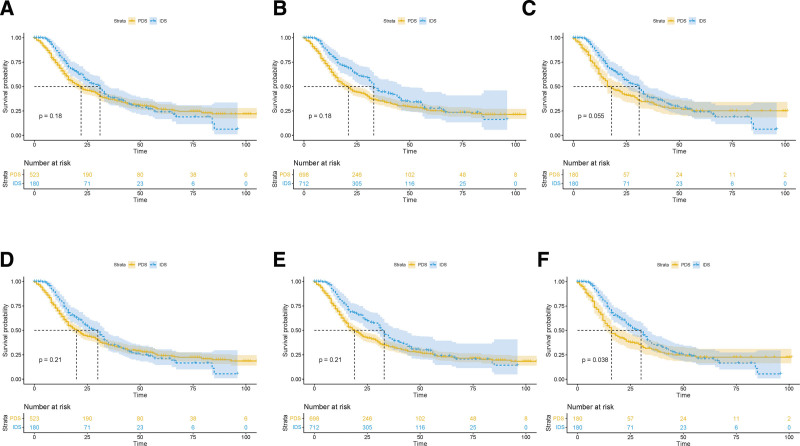
Kaplan–Meier survival curves of CSS and OS in unbalanced (A, D), IPTW-adjusted (B, E), and PSM (C, F) cohorts of patients receiving NACT or PDS in carcinosarcoma. CSS = cause-specific survival, IPTW = inverse probability of treatment weight, NACT = neoadjuvant chemotherapy, OS = overall survival, PDS = primary debulking surgery, PSM = propensity score model.

Originally, patients with advanced ovarian carcinosarcoma in NACT group were older, suffering from bilateral disease and distant metastasis. PSM and IPTW well balanced baseline characteristics between groups (see Table S5, http://links.lww.com/MD/I395, Supplemental Digital Content). More patients in IDS group achieved complete resection in unbalanced cohort (40.9% vs 45.0%), IPTW cohort (39.2% vs 44.1%) and PSM cohort (37.8% vs 45.0%, Table S6, http://links.lww.com/MD/I396, Supplemental Digital Content). Compared with IDS, PDS showed no survival benefit either in the unbalanced cohort (OS 20 vs 30 months, *P* = .21; CSS 22 vs 31 months, *P* = .18) or IPTW cohort (OS 19 vs 32 months, *P* = .21; CSS 21 vs 33 months, *P* = .18). Interestingly, in PSM cohort, patients had a superior survival (OS 16 vs 30 months, *P* = .038; CSS 18 vs 31 months, *P* = .055, Fig. 4). Multivariate analysis identified NACT as a protective factor for OS in unbalanced cohort (HR 0.74; 95% CI 0.58–0.93; *P* = .010), IPTW cohort (HR 0.75; 95% CI 0.59–0.95; *P* = .019), and PSM cohort (HR 0.67; 95% CI 0.51–0.88; *P* = .004). The same result was observed in CSS (Table S7, http://links.lww.com/MD/I397, Supplemental Digital Content). In addition, age was identified as independent prognostic factor in all models.

### 3.5. Endometrioid carcinoma, mixed cell carcinoma, LGSC and mucinous carcinoma

For endometrioid carcinoma, more patients in NACT group had stage IV disease, elevated CA125 and distant metastasis (Table S8, http://links.lww.com/MD/I398, Supplemental Digital Content). The PDS group exhibited a significant better survival than IDS group in the unbalanced cohort (OS 77 vs 35 months, *P* = .003; CSS 81 vs 35 months, *P* = .020) and IPTW cohort (OS 73 vs 35 months, *P* = .003; CSS 81 vs 40 months, *P* = .020), while not in PSM cohort (OS 61 vs 35 months, *P* = .06; the median CSS times were not reached, *P* = .15, Fig. S2, http://links.lww.com/MD/I387, Supplemental Digital Content). Only in IPTW cohort, IDS was a risk factor for OS after adjusted for covariates (HR 1.48; 95% CI 1.00-2.19; *P* = .048, Table S9, http://links.lww.com/MD/I399, Supplemental Digital Content). In all cohorts, increased age and high-grade tumor were risk factors for OS and CSS, while complete resection was a protective factor for CSS.

Baseline characteristics of the 3 models for mixed cell carcinoma are shown in Table S10, http://links.lww.com/MD/I400, Supplemental Digital Content. Patients receiving NACT were older, with higher rates of bilateral primary tumor, stage IV disease, distant metastasis and elevated CA125. PDS showed a significant survival benefit over IDS in the unbalanced cohort (OS 49 vs 29 months, *P* = .006; CSS 52 vs 30 months, *P* = .015) and IPTW cohort (OS 46 vs 30 months, *P* = .006; CSS 49 vs 30 months, *P* = .015) while not in PSM cohort (OS 36 vs 29 months, *P* = .060; CSS 37 vs 30 months, *P* = .600, Fig. S3, http://links.lww.com/MD/I388, Supplemental Digital Content). On multivariable analysis, NACT was a risk factor for OS (HR 1.43; 95% CI 1.07–1.91; *P* = .016) and CSS (HR 1.44; 95% CI 1.07–1.94; *P* = .017) only in IPTW cohort (Table S11, http://links.lww.com/MD/I401, Supplemental Digital Content).,

IDS were more frequently used in stage IV LGSC patients (Table S12, http://links.lww.com/MD/I402, Supplemental Digital Content). PDS revealed a significant survival benefit over IDS in some of the models: unbalanced cohort (OS 90 vs 66 months, *P* = .086; the median CSS times were not reached, *P* = .043), IPTW cohort (CSS, *P* = .043; the median OS were not reached, *P* < .086) and PSM cohort (CSS, *P* = .075; OS, *P* = .15; the median OS and CSS were not reached, Fig. S4, http://links.lww.com/MD/I389, Supplemental Digital Content).

In mucinous carcinoma, women with distant metastasis were apt to receive IDS (Table S13, http://links.lww.com/MD/I403, Supplemental Digital Content). The survival of patients receiving IDS were non-inferior to PDS in unbalanced cohort (OS 18 vs 17 months, *P* = .96; CSS 22 vs 20 months, *P* = .94), IPTW cohort (OS 20 vs 10 months, *P* = .96; CSS 26 vs 10 months, *P* = .94) or PSM cohort (OS 7 vs 17 months, *P* = .32; CSS 10 vs 20 months, *P* = .35, Fig. S5, http://links.lww.com/MD/I390, Supplemental Digital Content). Multivariable analysis was not conducted in LGSC and mucinous carcinoma due to limited sample size.

## 4. Discussion

In recent years, NACT is increasingly being offered to advanced EOC patients when optimal debulking is difficult to achieve or there is a significant risk for surgical complication.^[4]^ Although several prospective randomized phase III trials demonstrated higher rates of optimal debulking, reduction of peri-operative complications and non-inferior prognosis of NACT, the validity of these trials is questioned due to deficiencies in study design and quality of surgery.^[5,7–9]^ However, when it comes to “real-world practice,” much of the retrospective observational researches revealed a consistently shorter survival time of NACT.^[23]^ The distinct results of randomized controlled trials and retrospective studies suggested that effect of NACT may depend on clinical feature of patient and tumor biology. Considering the importance of optimal debulking, additional attempts to define the best candidates for NACT mainly focused on clinical factors such as tumor load, comorbidities, performance status and age,^[18,24,25]^ while ignoring the intrinsic heterogeneity in biology.

As a family of related but distinct cancers with substantial differences in pathobiological feature, each subtype of EOC is associated with specific clinical behavior and chemotherapy response but has been treated as one entity.^[26,27]^ Most of the published data regarding NACT was primarily based on the predominant HGSC.^[28]^ The less common subtypes have been understudied due to their rarity, and it’s not yet clear whether patients with less common subtype can benefit from NACT. For these patients with limited valid treatment options, additional information from large sample retrospective studies is important, since it is difficult to acquire prospective data. This “real-world observational study” showed that HGSC patients receiving NACT represent a high-risk population with worse prognosis as compared to candidates for PDS.^[13,18,29]^ The results hold true after controlling for potential bias through propensity matching and weighted analyses, even after adjusting for potential prognostic covariates. Also, we observed a similar rate of patients achieving no gross residuals in the IPTW model and a slightly higher rate in the NACT cohort in PSM model. The reason for impaired long-term survival of NACT remains unclear.^[29]^ One biggest criticism for this is the possible emergence of drug-resistance,^[30–33]^ especially in HGSC. Mathematical framework developed by Shengqing et al showed that NACT significantly enriches chemotherapy resistant HGSC cells while killing the chemo-sensitive ones; by contrast, PDS can effectively remove resistant cells, leaving the following chemotherapy to deplete residual sensitive cells.^[34]^ What’s more, fibrosis and necrosis induced by NACT may interfere the evaluation and resection of tumor areas at PDS.^[35]^ The underestimation of tumor spread as well as incomplete resection of potentially resectable tumor areas are likely to have an unfavorable effect on patient outcome. Except for the possible explanation of chemotherapy resistance and misjudgment of residual disease, the results of this observational study could be attributed to other unmeasured factors, such as surgical approaches, comorbidities, BRCA status and maintenance therapy.^[36–39]^

Primary ovarian carcinosarcomas are highly aggressive tumors with both carcinomatous and sarcomatous components that usually diagnosed at advanced stage (75%–80%).^[40,41]^ Primary treatment guidelines for this subtype extrapolated from HGSC has traditionally been PDS followed by chemotherapy and/or radiotherapy.^[42]^ No current evidence to guide clinical practice regards to NACT. However, different from HGSC, a superior OS and CSS was observed among patients receiving NACT, in both original cohorts and adjusted cohorts. Different chemo-responsiveness and tumor visibility between HGSC and carcinosarcoma after NACT provided a reasonable explanation. HGSC is quite chemo-responsive while carcinosarcoma usually presents chemoresistance.^[42]^ Reduction of tumor volume after NACT appears to be less significant for carcinosarcoma, leading to better lesion visibility during IDS, especially for those small foci that are likely to be omitted in HGSC. More accurate judgement for residual disease after debulking may underly the different outcomes of 2 subtypes. This also works for clear cell carcinoma. Quite contrary to the current opinion that PDS provides the best treatment option for advanced clear cell carcinoma,^[43,44]^ our result demonstrated a survival benefit from NACT.

In the subset of patients with endometrioid carcinoma and mixed cell carcinoma, NACT resulted in non-significant inferior trend towards OS and CSS as compared with PDS. Endometrioid carcinoma takes up for 10% of ovarian cancer, with the large proportion of patients diagnosed at low-grade, early disease.^[45]^ Notably, nearly half of the advanced endometrioid carcinoma patients in our study exhibited high-grade disease, and experienced worse survival in contrast to their low-grade counterparts. In fact, misclassification of serous carcinoma as endometrioid carcinoma is documented^[46]^; and it has been postulated that the majority of high-grade endometrioid carcinomas represent serous carcinomas with variant morphology.^[47]^ Further stratified analysis of NACT based on a more accurate pathology may provide particularly valuable information for this subset. Mixed cell ovarian carcinoma are defined when 10 % or more of a tumor show any other line of differentiation.^[48]^ Since current literatures reporting mixed cell carcinoma are mainly individual cases, with unclear clinical characteristics and prognosis.^[49]^ Although a retrospective study based on SEER database suggested that age, grade and stage were potential risk factors for mixed cell carcinoma,^[49]^ they didn’t involve NACT. Our study showed that NACT may associated with inferior prognosis in advance ovarian mixed cell carcinoma. Results of LGSC and mucinous carcinoma should be discussed cautiously, since the subgroups of patients with LGSC and mucinous carcinoma was too small for meaningful analysis. Current evidences support that cytoreduction remains the most important prognostic factor due to limited efficacy of chemotherapy in this cluster of patients.^[50–54]^

Notably, disparate survival was reported when applying IPTW versus PSM to assess outcomes of different treatments. In PSM analysis, although the exclusion of low-scored cases decreased the model dependence, the interpretation of results was much closer to “real-world practice.”^[55]^ Specifically, patients with lower propensity scores tended to have better prognosis because they were more likely to have less risk factors for PDS. On the other hand, the IPTW provided an ideal counterfactual scenario where everyone was offered the assigned treatment, which may not play out in the real world. What’s more, in small sample cohorts, the weighting results in a significant distortion of population.^[56]^ This was also illustrated in several of our subsets, where the sample size after IPTW was far above the actual sample size. To this point, PSM model was therefore more appropriate in our smaller sample cohorts.

## 5. Strengths and limitations

Strengths of the current study include a large sample size and propensity score weighting for background adjustment to assess ovarian cancer mortality. However, some limitations could not be overlooked. First, the program does not have information for recurrence, surgical complications and side-effects of chemotherapy, which are the salient factors when considering NACT. Second, missing data on tumor volume, grade and residual disease might cause selective bias. Third, the lacking detailed information on important prognostic factors such as chemotherapy regimen and cycles, BRCA mutation status and maintenance therapy precluded our ability to evaluate confounding factors. Also, central pathology reviews of the histology were not available for patients with EOC who were registered in the SEER program. Last, even SEER database provided a large cohort size, the number of patients with rare subtype (mucinous carcinoma and LGSC) was still too small and limited our power to come to any formal conclusion.

## 6. Conclusion

Overall, our study substantiated previous findings showing that therapeutic effect of NACT on advanced EOC differed from pathological subtypes. Although an inferior prognosis of NACT in HGSC was indicated, patients with less common histo-subtypes such as clear cell carcinoma and carcinosarcoma may benefit from NACT. In light of this results, further prospective research assessing the effect of NACT with different types of EOC is warranted.

## Author contributions

**Conceptualization:** Yuexi Liu, Qiuying Gu, Yao Xiao, Xinyue Du.

**Data curation:** Yuexi Liu, Meng Ni.

**Formal analysis:** Meng Ni.

**Methodology:** Yuexi Liu, Meng Ni, Fanfan Huang, Yao Xiao.

**Resources:** Yuexi Liu.

**Software:** Meng Ni.

**Supervision:** Yuexi Liu.

**Writing – original draft:** Yuexi Liu, Meng Ni, Fanfan Huang.

**Writing – review & editing:** Yuexi Liu, Meng Ni, Fanfan Huang, Qiuying Gu, Yao Xiao, Xinyue Du.

## Supplementary Material
