# Supplementary material for: Neoadjuvant chemotherapy in advanced epithelial ovarian cancer by histology: A SEER based survival analysis
Source: Medicine (Baltimore). 2023 Jan 27;102(4):e32774. doi: 10.1097/MD.0000000000032774 (PMC9875958; doi:10.1097/MD.0000000000032774)

Supplementary Figure S1 Use of NACT versus PDS for advanced epithelial ovarian cancer over time from the SEER database, 2010-2018.

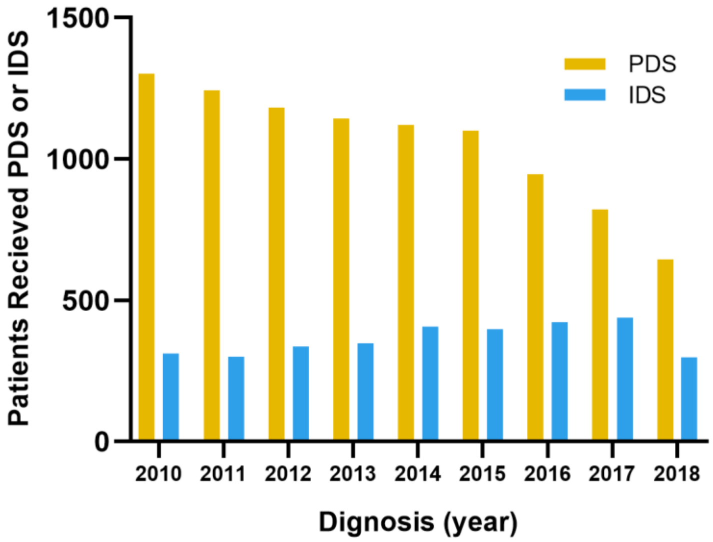

Supplement: Supplementary file 1 [file medi-102-e32774-s001.pdf]
