# Supplementary material for: Neoadjuvant chemotherapy in advanced epithelial ovarian cancer by histology: A SEER based survival analysis
Source: Medicine (Baltimore). 2023 Jan 27;102(4):e32774. doi: 10.1097/MD.0000000000032774 (PMC9875958; doi:10.1097/MD.0000000000032774)
Supplement: Supplementary file 2 [file medi-102-e32774-s002.pdf]

Table S1. Demographics of patients with advanced HGSC in the unbalanced, IPTW and PSM population

Characteristics		Unbalanced Population, N (%)			IPTW, N (%)			PSM, N (%)			
		PDS<br>N = 7009	IDS<br>N = 2729	*p*-value	PDS<br>N = 9754	IDS<br>N= 9660	*p*-value	PDS<br>N= 2729	IDS<br>N= 2729	*p*-value	SMD
Age, mean (SD)		61.14 (11.53)	63.79 (10.81)	<0.001	61.88 (11.43)	62.04 (11.05)	0.59	63.18 (11.05)	63.79 (10.81)	0.038	0.056
Race	White	5929 (84.6)	2248 (82.4)	0.048	8204.6 (84.1)	8115.1 (84.0)	0.911	2241 (82.1)	2248 (82.4)	0.896	0.021
	Black	470 (6.7)	220 (8.1)		690.8 (7.1)	723.6 (7.5)		223 (8.2)	220 (8.1)		
	Others	597 (8.5)	256 (9.4)		840.2 (8.6)	802.8 (8.3)		262 (9.6)	256 (9.4)		
	Unknown	13 (0.2)	5 (0.2)		18.1 (0.2)	18.5 (0.2)		3 (0.1)	5 (0.2)		
Marriage	Single	2661 (38.0)	1157 (42.4)	<0.001	3825.7 (39.2)	3806.0 (39.4)	0.981	1128 (41.3)	1157 (42.4)	0.328	0.04
	Married	4071 (58.1)	1473 (54.0)		5551.8 (56.9)	5475.9 (56.7)		1482 (54.3)	1473 (54.0)		
	Unknown	277 (4.0)	99 (3.6)		376.2 (3.9)	378.0 (3.9)		119 (4.4)	99 (3.6)		
Grade	G1-G2	574 (8.2)	128 (4.7)	<0.001	707.9 (7.3)	704.8 (7.3)	0.961	148 (5.4)	128 (4.7)	0.241	0.033
	G3-G4	6435 (91.8)	2601 (95.3)		9045.9 (92.7)	8955.1 (92.7)		2581 (94.6)	2601 (95.3)		
	Unknown	-	-		-	-		-	-		
Laterality	Unilateral	2546 (36.3)	936 (34.3)	0.064	3474.7 (35.6)	3477.6 (36.0)	0.765	899 (32.9)	936 (34.3)	0.302	0.029
	Bilateral	4463 (63.7)	1793 (65.7)		6279.0 (64.4)	6182.3 (64.0)		1830 (67.1)	1793 (65.7)		
FIGO stage	IIIA	239 (3.4)	60 (2.2)	<0.001	292.2 (3.0)	228.0 (2.4)	0.552	51 (1.9)	60 (2.2)	0.729	0.039
	IIIB	497 (7.1)	103 (3.8)		596.7 (6.1)	551.7 (5.7)		108 (4.0)	103 (3.8)		
	IIIC	4246 (60.6)	1103 (40.4)		5346.0 (54.8)	5336.0 (55.2)		1139 (41.7)	1103 (40.4)		
	IIINOS	321 (4.6)	163 (6.0)		481.4 (4.9)	467.4 (4.8)		168 (6.2)	163 (6.0)		
	IV	1706 (24.3)	1300 (47.6)		3037.4 (31.1)	3076.8 (31.9)		1263 (46.3)	1300 (47.6)		
Pretreatment CA125	Normal/negative	245 (3.5)	41 (1.5)		285.1 (2.9)	278.3 (2.9)	0.975	33 (1.2)	41 (1.5)	0.044	0.068
	Elevated/positive	5761 (82.2)	2454 (89.9)	<0.001	8232.9 (84.4)	8177.8 (84.7)		2411 (88.3)	2454 (89.9)		

	Unknown	1003 (14.3)	234 (8.6)		1235.7 (12.7)	1203.8 (12.5)		285 (10.4)	234 (8.6)		
Tumor volume	≤10cm	3929 (56.1)	1308 (47.9)	<0.001	5242.6 (53.7)	5266.7 (54.5)	0.769	1385 (50.8)	1308 (47.9)	0.005	0.088
	＞10cm	2026 (28.9)	402 (14.7)		2425.0 (24.9)	2330.8 (24.1)		439 (16.1)	402 (14.7)		
	Unknown	1054 (15.0)	1019 (37.3)		2086.1 (21.4)	2062.4 (21.4)		905 (33.2)	1019 (37.3)		
Distant metastasis (brain/lung/bone/liver)	No	6485 (92.5)	2222 (81.4)	<0.001	8694.6 (89.1)	8602.7 (89.1)	0.902	2273 (83.3)	2222 (81.4)	0.076	0.049
	Yes	524 (7.5)	507 (18.6)		1059.1 (10.9)	1057.2 (10.9)		456 (16.7)	507 (18.6)		
Radiation	No/unknown	6948 (99.1)	2706 (99.2)	0.992	9670.8 (99.1)	9580.5 (99.2)	0.897	2706 (99.2)	2706 (99.2)	1	<0.001
	Yes	61 (0.9)	23 (0.8)		82.9 (0.9)	79.4 (0.8)		23 (0.8)	23 (0.8)		
