# Supplementary material for: Neoadjuvant chemotherapy in advanced epithelial ovarian cancer by histology: A SEER based survival analysis
Source: Medicine (Baltimore). 2023 Jan 27;102(4):e32774. doi: 10.1097/MD.0000000000032774 (PMC9875958; doi:10.1097/MD.0000000000032774)
Supplement: Supplementary file 3 [file medi-102-e32774-s003.pdf]

Table S2. Multivariable analysis of risk factor of the patients with serous high-grade carcinoma

**OS**										
Characteristics		Unbalanced Population			IPTW			PSM		
		HR	95%CI	*p*-value	HR	95%CI	*p*-value	HR	95%CI	*p*-value
NACT	PDS	Reference			Reference			Reference		
	IDS	1.3	1.22-1.39	<0.001***	1.34	1.25-1.44	<0.001***	1.26	1.17-1.36	<0.001***
Age, mean (SD)		1.02	1.02-1.02	<0.001***	1.02	1.01-1.02	<0.001***	1.02	1.01-1.02	<0.001***
Race	White	Reference			Reference			Reference		
	Black	1.13	1.02-1.26	0.025*	1.09	0.96-1.25	0.193	1.11	0.97-1.27	0.116
	Others	0.9	0.81-1.00	0.045*	0.89	0.78-1.02	0.086	0.8	0.69-0.91	0.001**
	Unknown	0.47	0.12-1.87	0.282	0.46	0.09-2.26	0.337	0.26	0.04-1.85	0.179
Marriage	Single	Reference			Reference			Reference		
	Married	0.85	0.80-0.91	<0.001***	0.87	0.80-0.93	<0.001***	0.89	0.83-0.96	0.003**
	Unknown	0.91	0.79-1.06	0.241	0.82	0.68-0.99	0.039*	0.88	0.73-1.06	0.173
Grade	G1-G2	Reference			Reference			Reference		
	G3-G4	1.03	0.93-1.15	0.558	0.97	0.85-1.12	0.71	1.03	0.88-1.21	0.69
	Unknown	-	-	-	-	-	-	-	-	-
Laterality	Unilateral	Reference			Reference			Reference		
	Bilateral	1.18	1.11-1.25	<0.001***	1.13	1.05-1.23	0.001**	1.16	1.07-1.26	<0.001***
FIGO stage	IIIA	Reference			Reference			Reference		
	IIIB	1.04	0.81-1.34	0.749	1.08	0.80-1.46	0.628	1.24	0.84-1.82	0.288
	IIIC	1.48	1.19-1.84	<0.001***	1.47	1.15-1.88	0.002**	1.65	1.18-2.30	0.003**
	IIINOS	1.51	1.19-1.93	<0.001***	1.53	1.16-2.01	0.003**	1.59	1.11-2.27	0.011*
	IV	1.78	1.43-2.23	<0.001***	1.75	1.36-2.25	<0.001***	1.87	1.34-2.61	<0.001***
Pretreatment CA125	Normal/negative	Reference			Reference			Reference		
	Elevated/positive	1.48	1.21-1.81	<0.001***	1.5	1.18-1.91	<0.001***	1.09	0.80-1.49	0.566
	Unknown	1.44	1.16-1.78	<0.001***	1.39	1.06-1.81	0.016*	1.04	0.75-1.44	0.821
Tumor volume	≤10cm	Reference			Reference			Reference		
	＞10cm	0.82	0.76-0.89	<0.001***	0.84	0.76-0.93	<0.001***	0.84	0.75-0.93	0.001**
	Unknown	1.05	0.98-1.13	0.169	1.07	0.99-1.16	0.072	1.03	0.95-1.11	0.492
	No	Reference			Reference			Reference		

Distant metastasis (brain/lung/bone/liver)	Yes	1.06	0.96-1.18	0.229	1.07	0.96-1.20	0.215	1.07	0.97-1.19	0.188
Radiation	No	Reference			Reference			Reference		
	Yes	1.39	1.07-1.81	0.014*	1.34	1.01-1.77	0.044*	1.35	0.97-1.87	0.077
Surgery outcome	R0	Reference			Reference			Reference		
	None R0	1.57	1.47-1.68	<0.001***	1.52	1.40-1.65	<0.001***	1.45	1.33-1.59	<0.001***
	Unknown	1.35	1.24-1.48	<0.001***	1.35	1.21-1.50	<0.001***	1.3	1.16-1.46	<0.001***

CSS

Characteristics		Unbalanced Population			IPTW			PSM		
		HR	95%CI	*p*-value	HR	95%CI	*p*-value	HR	95%CI	*p*-value
NACT	PDS	Reference			Reference			Reference		
	IDS	1.33	1.24-1.42	<0.001***	1.37	1.27-1.47	<0.001***	1.29	1.20-1.39	<0.001***
Age, mean (SD)		1.02	1.01-1.02	<0.001***	1.02	1.01-1.02	<0.001***	1.02	1.01-1.02	<0.001***
Race	White	Reference			Reference			Reference		
	Black	1.12	1.00-1.25	0.051	1.1	0.96-1.27	0.171	1.11	0.97-1.27	0.139
	Others	0.91	0.81-1.01	0.083	0.91	0.80-1.04	0.187	0.81	0.71-0.94	0.004**
	Unknown	0.51	0.13-2.03	0.336	0.49	0.10-2.45	0.384	0.28	0.04-2.02	0.208
Marriage	Single	Reference			Reference			Reference		
	Married	0.86	0.81-0.92	<0.001***	0.88	0.81-0.94	<0.001***	0.9	0.84-0.97	0.009**
	Unknown	0.92	0.79-1.07	0.282	0.82	0.67-0.99	0.043*	0.88	0.72-1.07	0.191
Grade	G1-G2	Reference			Reference			Reference		
	G3-G4	1.02	0.92-1.14	0.689	0.96	0.83-1.10	0.532	1.03	0.87-1.20	0.753
	Unknown	-	-	-	-	-	-	-	-	-
Laterality	Unilateral	Reference			Reference			Reference		
	Bilateral	1.19	1.11-1.26	<0.001***	1.14	1.05-1.24	0.001**	1.17	1.08-1.27	<0.001***
FIGO stage	IIIA									
	IIIB	1.03	0.79-1.35	0.810144	1.04	0.75-1.43	0.833	1.1	0.73-1.64	0.65
	IIIC	1.54	1.22-1.93	<0.001***	1.49	1.15-1.93	0.002**	1.57	1.12-2.20	0.009**
	IIINOS	1.54	1.19-1.99	0.001 **	1.52	1.14-2.03	0.004**	1.47	1.02-2.12	0.037*
	IV	1.84	1.46-2.32	<0.001***	1.76	1.36-2.29	<0.001***	1.76	1.26-2.48	0.001**

	Normal/negative	Reference			Reference			Reference		
Pretreatment CA125	Elevated/positive	1.45	1.18-1.78	<0.001***	1.46	1.14-1.86	0.003**	1.1	0.80-1.52	0.549
	Unknown	1.4	1.12-1.74	0.003**	1.35	1.03-1.77	0.030*	1.03	0.73-1.44	0.885
	≤10cm	Reference			Reference			Reference		
Tumor volume	＞10cm	0.83	0.77-0.89	<0.001***	0.84	0.76-0.93	0.001**	0.85	0.76-0.95	0.004**
	Unknown	1.05	0.98-1.13	0.198	1.08	0.99-1.17	0.071	1.03	0.95-1.12	0.481
Distant metastasis	No	Reference			Reference			Reference		
(brain/lung/bone/liver)	Yes	1.07	0.97-1.19	0.175	1.08	0.96-1.21	0.179	1.09	0.97-1.21	0.135
Radiation	No	Reference			Reference			Reference		
	Yes	1.42	1.09-1.86	0.010*	1.37	1.02-1.83	0.034*	1.39	1.00-1.95	0.052
	R0	Reference			Reference			Reference		
Surgery outcome	None R0	1.62	1.51-1.74	<0.001***	1.56	1.44-1.70	<0.001***	1.51	1.37-1.66	<0.001***
	Unknown	1.39	1.27-1.52	<0.001***	1.37	1.23-1.54	<0.001***	1.34	1.19-1.50	<0.001***
