# Supplementary material for: Neoadjuvant chemotherapy in advanced epithelial ovarian cancer by histology: A SEER based survival analysis
Source: Medicine (Baltimore). 2023 Jan 27;102(4):e32774. doi: 10.1097/MD.0000000000032774 (PMC9875958; doi:10.1097/MD.0000000000032774)
Supplement: Supplementary file 4 [file medi-102-e32774-s004.pdf]

Table S3. Demographics of patients with clear cell carcinoma in the unbalanced, IPTW and PSM population

Characteristics		Unbalanced Population, N (%)			IPTW, N (%)			PSM, N (%)		
		PDS N = 442	IDS N = 93	*p*-value	PDS N = 539	IDS N= 482	*p*-value	PDS N= 93	IDS N= 93	*p*-value
Age, mean (SD)		56.36 (10.56)	60.42 (10.66)	0.001	57.26 (10.87)	58.79 (11.00)	0.369	59.94 (11.26)	60.42 (10.66)	0.764
Race	White	321 (72.6)	70 (75.3)		392.8 (72.9)	346.0 (71.8)		68 (73.1)	70 (75.3)	
	Black	26 (5.9)	7 (7.5)	0.744	34.2 (6.3)	29.9 (6.2)	0.919	7 (7.5)	7 (7.5)	0.929
	Others	94 (21.3)	16 (17.2)		111.1 (20.6)	106.2 (22.0)		18 (19.4)	16 (17.2)	
	Unknown	1 (0.2)	0 (0.0)		1.0 (0.2)	0.0 (0.0)		0.0 (0.0)	0.0 (0.0)	
Marriage	Single	186 (42.1)	45 (48.4)	0.418	235.8 (43.7)	223.9 (46.4)	0.534	43 (46.2)	45 (48.4)	0.789
	Married	238 (53.8)	46 (49.5)		283.7 (52.6)	251.5 (52.2)		49 (52.7)	46 (49.5)	
	Unknown	18 (4.1)	2 (2.2)		19.6 (3.6)	6.7 (1.4)		1 (1.1)	2 (2.2)	
Grade	G1-G2	23 (5.2)	2 (2.2)	0.035	25.1 (4.7)	20.7 (4.3)	0.987	2 (2.2)	2 (2.2)	0.989
	G3-G4	269 (60.9)	47 (50.5)		316.6 (58.7)	280.3 (58.1)		48 (51.6)	47 (50.5)	
	Unknown	150 (33.9)	44 (47.3)		197.4 (36.6)	181.1 (37.6)		43 (46.2)	44 (47.3)	
Laterality	Unilateral	309 (69.9)	46 (49.5)	<0.001	357.2 (66.3)	301.6 (62.6)	0.602	56 (60.2)	46 (49.5)	0.185
	Bilateral	133 (30.1)	47 (50.5)		181.9 (33.7)	180.5 (37.4)		37 (39.8)	47 (50.5)	
FIGO stage	IIIA	48 (10.9)	4 (4.3)	<0.001	52.1 (9.7)	48.2 (10.0)	0.967	2 (2.2)	4 (4.3)	0.926
	IIIB	39 (8.8)	4 (4.3)		42.8 (7.9)	36.5 (7.6)		4 (4.3)	4 (4.3)	
	IIIC	250 (56.6)	26 (28.0)		275.4 (51.1)	225.9 (46.9)		24 (25.8)	26 (28.0)	
	IIINOS	18 (4.1)	8 (8.6)		27.9 (5.2)	32.4 (6.7)		9 (9.7)	8 (8.6)	
	IV	87 (19.7)	51 (54.8)		140.9 (26.1)	139.2 (28.9)		54 (58.1)	51 (54.8)	
Pretreatment CA125	Normal/negative	42 (9.5)	2 (2.2)	0.009	44.1 (8.2)	36.4 (7.6)	0.691	2 (2.2)	2 (2.2)	0.799
	Elevated/positive	322 (72.9)	81 (87.1)		407.4 (75.6)	391.0 (81.1)		78 (83.9)	81 (87.1)	
	Unknown	78 (17.6)	10 (10.8)		87.6 (16.3)	54.7 (11.3)		13 (14.0)	10 (10.8)	
Tumor volume	≤10cm	136 (30.8)	40 (43.0)	<0.001	181.9 (33.7)	174.4 (36.2)	0.891	43 (46.2)	40 (43.0)	0.833
	＞10cm	262 (59.3)	34 (36.6)		294.9 (54.7)	248.8 (51.6)		34 (36.6)	34 (36.6)	

	Unknown	44 (10.0)	19 (20.4)		62.4 (11.6)	59.0 (12.2)		16 (17.2)	19 (20.4)	
Distant metastasis (brain/lung/ bone/liver)	No	404 (91.4)	74 (79.6)	0.001	481.0 (89.2)	421.7 (87.5)	0.629	71 (76.3)	74 (79.6)	0.724
	Yes	38 (8.6)	19 (20.4)		58.1 (10.8)	60.4 (12.5)		22 (23.7)	19 (20.4)	
Radiation	No/unknown	435 (98.4)	91 (97.8)	1	530.6 (98.4)	465.4 (96.5)	0.314	90 (96.8)	91 (97.8)	1
	Yes	7 (1.6)	2 (2.2)		8.5 (1.6)	16.7 (3.5)		3 (3.2)	2 (2.2)	
