# Supplementary material for: Neoadjuvant chemotherapy in advanced epithelial ovarian cancer by histology: A SEER based survival analysis
Source: Medicine (Baltimore). 2023 Jan 27;102(4):e32774. doi: 10.1097/MD.0000000000032774 (PMC9875958; doi:10.1097/MD.0000000000032774)
Supplement: Supplementary file 5 [file medi-102-e32774-s005.pdf]

Table S4. Multivariable analysis of risk factor of the patients with clear cell carcinoma

OS										
Characteristics		Unbalanced Population			IPTW			PSM		
		HR	95%CI	*p*-value	HR	95%CI	*p*-value	HR	95%CI	*p*-value
NACT	PDS	Reference			Reference			Reference		
	IDS	0.57	0.40-0.82	0.002**	0.77	0.45-1.31	0.337	0.5	0.33-0.76	0.001**
Age, mean (SD)		0.99	0.98-1.00	0.066	1	0.98-1.02	0.972	0.99	0.97-1.01	0.397
Race	White	Reference			Reference			Reference		
	Black	1.52	0.93-2.48	0.092	1.49	0.88-2.50	0.134	1.56	0.68-3.55	0.292
	Others	0.92	0.68-1.24	0.596	0.79	0.51-1.23	0.298	0.91	0.53-1.55	0.724
	Unknown	-	-	0.991	-	-	<0.001***	NA	NA	NA
Marriage	Single	Reference			Reference			Reference		
	Married	1.32	1.03-1.69	0.028*	1.66	1.18-2.33	0.003**	1.71	1.12-2.62	0.013*
	Unknown	1	0.50-2.01	0.995	1	0.42-2.37	0.993	1.46	0.33-6.53	0.622
Grade	G1-G2	Reference			Reference			Reference		
	G3-G4	1.16	0.65-2.06	0.626	1.59	0.82-3.10	0.173	2.69	0.35-20.58	0.341
	Unknown	1.02	0.56-1.85	0.952	1.53	0.76-3.07	0.231	2.39	0.31-18.48	0.404
Laterality	Unilateral	Reference			Reference			Reference		
	Bilateral	1.58	1.24-2.03	<0.001***	1.69	1.15-2.50	0.008**	1.69	1.12-2.56	0.013*
FIGO stage	IIIA	Reference			Reference			Reference		
	IIIB	1.03	0.57-1.87	0.926	1.33	0.49-3.59	0.577	0.78	0.16-3.89	0.765
	IIIC	1.05	0.67-1.63	0.843	0.86	0.40-1.83	0.699	0.83	0.24-2.90	0.772
	IIINOS	0.75	0.37-1.51	0.418	0.59	0.25-1.40	0.228	0.63	0.16-2.50	0.51
	IV	1.48	0.88-2.50	0.144	1.03	0.46-2.32	0.947	1.01	0.29-3.50	0.987
Pretreatment CA125	Normal/negative	Reference			Reference			Reference		
	Elevated/positive	1.61	0.99-2.60	0.055	0.83	0.29-2.35	0.718	0.82	0.19-3.53	0.788
	Unknown	1.14	0.67-1.96	0.627	0.58	0.21-1.64	0.307	0.33	0.07-1.68	0.184
Tumor volume	≤10cm	Reference			Reference			Reference		

	＞10cm	0.77	0.60-1.00	0.053	0.92	0.61-1.38	0.675	0.61	0.38-0.99	0.044*
	Unknown	0.81	0.54-1.22	0.307	0.72	0.44-1.19	0.206	0.76	0.43-1.35	0.354
Distant metastasis (brain/lung/bone/liver)	No	Reference			Reference			Reference		
	Yes	1.17	0.75-1.84	0.493	1.21	0.73-2.00	0.456	1.14	0.67-1.95	0.624
Radiation	No	Reference			Reference			Reference		
	Yes	1.11	0.39-3.19	0.848	1.3	0.50-3.33	0.592	2.41	0.69-8.38	0.166
Surgery outcomes	R0	Reference			Reference			Reference		
	None R0	1.58	1.21-2.06	<0.001***	1.49	1.01-2.19	0.045*	1.27	0.77-2.10	0.356
	Unknown	1.3	0.92-1.83	0.133	1.72	0.95-3.09	0.072	1.89	1.03-3.47	0.040*

**CSS**										
Characteristics		Unbalanced Population			IPTW			PSM		
		HR	95%CI	*p*-value	HR	95%CI	*p*-value	HR	95%CI	*p*-value
NACT	PDS	Reference			Reference			Reference		
	IDS	0.61	0.42-0.88	0.009**	0.75	0.45-1.26	0.277	0.52	0.34-0.79	0.002**
Age, mean (SD)		0.99	0.98-1.00	0.038*	1	0.98-1.01	0.691	0.99	0.97-1.01	0.393
Race	White	Reference			Reference			Reference		
	Black	1.48	0.89-2.44	0.129	1.45	0.86-2.46	0.164	1.54	0.67-3.53	0.31
	Others	0.89	0.65-1.23	0.493	0.66	0.44-0.99	0.046*	0.79	0.45-1.41	0.429
	Unknown [a]	-	-	0.991	-	-	<0.001***	NA	NA	NA
Marriage	Single	Reference			Reference			Reference		
	Married	1.3	1.00-1.67	0.048*	1.57	1.13-2.18	0.007**	1.65	1.07-2.56	0.025*
	Unknown	0.96	0.46-2.00	0.913	0.96	0.39-2.35	0.926	1.46	0.32-6.58	0.621
Grade	G1-G2	Reference			Reference			Reference		
	G3-G4	1.27	0.68-2.37	0.456	1.7	0.85-3.41	0.135	2.36	0.31-18.21	0.411
	Unknown	1.08	0.57-2.05	0.819	1.46	0.71-2.98	0.305	2.1	0.27-16.41	0.481
Laterality	Unilateral	Reference			Reference			Reference		
	Bilateral	1.68	1.29-2.17	<0.001***	1.87	1.26-2.78	0.002**	1.79	1.16-2.75	0.008**

	IIIA	Reference			Reference			Reference		
	IIIB	0.84	0.44-1.62	0.605	0.74	0.30-1.82	0.507	0.6	0.11-3.34	0.562
FIGO stage	IIIC	1.02	0.64-1.62	0.928	0.87	0.38-1.99	0.746	0.86	0.25-3.03	0.821
	IIINOS	0.79	0.38-1.61	0.511	0.62	0.25-1.55	0.304	0.69	0.17-2.74	0.595
	IV	1.47	0.85-2.53	0.17	1.02	0.41-2.48	0.974	1.05	0.30-3.69	0.936
	Normal/negative	Reference			Reference			Reference		
Pretreatment CA125	Elevated/positive	1.46	0.89-2.41	0.134	0.78	0.28-2.17	0.628	0.78	0.18-3.38	0.737
	Unknown	1.15	0.66-2.00	0.627	0.59	0.21-1.64	0.309	0.35	0.07-1.76	0.201
	≤10cm	Reference			Reference			Reference		
Tumor volume	＞10cm	0.8	0.61-1.05	0.103	0.89	0.59-1.35	0.573	0.53	0.32-0.88	0.014*
	Unknown	0.93	0.61-1.41	0.725	0.81	0.49-1.34	0.415	0.75	0.42-1.34	0.338
Distant metastasis (brain/lung/bone/liver)	No	Reference			Reference			Reference		
	Yes	1.17	0.73-1.86	0.516	1.29	0.76-2.18	0.347	1.22	0.70-2.12	0.487
Radiation	No	Reference			Reference			Reference		
	Yes	1.22	0.42-3.52	0.711	1.39	0.56-3.48	0.481	2.62	0.74-9.21	0.134
	R0	Reference			Reference			Reference		
Surgery outcomes	None R0	1.57	1.19-2.08	0.001**	1.63	1.09-2.45	0.018*	1.33	0.78-2.25	0.293
	Unknown	1.26	0.88-1.80	0.213	1.95	1.08-3.52	0.026*	2.09	1.11-3.93	0.022*
