# Supplementary material for: Neoadjuvant chemotherapy in advanced epithelial ovarian cancer by histology: A SEER based survival analysis
Source: Medicine (Baltimore). 2023 Jan 27;102(4):e32774. doi: 10.1097/MD.0000000000032774 (PMC9875958; doi:10.1097/MD.0000000000032774)
Supplement: Supplementary file 6 [file medi-102-e32774-s006.pdf]

Table S5. Demographics of patients with carcinosarcoma in the unbalanced, IPTW and PSM population

Characteristics		Unbalanced Population, N (%)			IPTW, N (%)			PSM, N (%)		
		PDS<br>N = 553	IDS<br>N = 180	*p*-value	PDS<br>N = 698	IDS<br>N= 712	*p*-value	PDS<br>N= 180	IDS<br>N= 180	*p*-value
Age, mean (SD)		64.65 (10.80)	66.49 (10.44)	0.048	65.13 (10.67)	65.47 (10.20)	0.727	66.52 (10.71)	66.49 (10.44)	0.976
Race	White	443 (84.7)	150 (83.3)		588.4 (84.3)	612.4 (86.0)		149 (82.8)	150 (83.3)	
	Black	41 (7.8)	16 (8.9)	0.522	57.4 (8.2)	52.4 (7.4)	0.483	17 (9.4)	16 (8.9)	0.983
	Others	34 (6.5)	14 (7.8)		47.3 (6.8)	47.1 (6.6)		14 (7.8)	14 (7.8)	
	Unknown	5 (1.0)	0 (0.0)		5.0 (0.7)	0.0 (0.0)		-	-	
Marriage	Single	213 (40.7)	78 (43.3)	0.826	293.1 (42.0)	323.3 (45.4)	0.735	87 (48.3)	78 (43.3)	0.628
	Married	291 (55.6)	96 (53.3)		379.8 (54.4)	359.0 (50.4)		88 (48.9)	96 (53.3)	
	Unknown	19 (3.6)	6 (3.3)		25.2 (3.6)	29.6 (4.2)		5 (2.8)	6 (3.3)	
Laterality	Unilateral	319 (61.0)	90 (50.0)	<0.001	410.2 (58.8)	420.4 (59.1)	0.949	105 (58.3)	90 (50.0)	0.139
	Bilateral	204 (39.0)	90 (50.0)		288.0 (41.2)	291.5 (40.9)		75 (41.7)	90 (50.0)	
FIGO stage	IIIA	16 (3.1)	2 (1.1)		17.8 (2.5)	8.6 (1.2)		1 (0.6)	2 (1.1)	
	IIIB	41 (7.8)	8 (4.4)	<0.001	48.8 (7.0)	46.0 (6.5)	0.873	6 (3.3)	8 (4.4)	0.924
	IIIC	325 (62.1)	75 (41.7)		401.5 (57.5)	423.5 (59.5)		81 (45.0)	75 (41.7)	
	IIINOS	22 (4.2)	10 (5.6)		31.0 (4.4)	28.8 (4.0)		10 (5.6)	10 (5.6)	
	IV	119 (22.8)	85 (47.2)		199.1 (28.5)	205.0 (28.8)		82 (45.6)	85 (47.2)	
Pretreatment CA125	Normal/negative	30 (5.7)	5 (2.8)	0.116	34.7 (5.0)	38.1 (5.4)	0.884	4 (2.2)	5 (2.8)	0.85
	Elevated/positive	413 (79.0)	154 (85.6)		557.9 (79.9)	552.0 (77.5)		152 (84.4)	154 (85.6)	
	Unknown	80 (15.3)	21 (11.7)		105.6 (15.1)	121.8 (17.1)		24 (13.3)	21 (11.7)	
Tumor volume	≤10cm	174 (33.3)	79 (43.9)	<0.001	249.9 (35.8)	250.8 (35.2)	0.949	70 (38.9)	79 (43.9)	0.302
	＞10cm	280 (53.5)	56 (31.1)		338.8 (48.5)	354.8 (49.8)		70 (38.9)	56 (31.1)	
	Unknown	69 (13.2)	45 (25.0)		109.5 (15.7)	106.3 (14.9)		40 (22.2)	45 (25.0)	
Distant metastasis	No	480 (91.8)	136 (75.6)	<0.001	616.1 (88.2)	629.4 (88.4)	0.945	145 (80.6)	136 (75.6)	0.308
	Yes	43 (8.2)	44 (24.4)		82.1 (11.8)	82.5 (11.6)		35 (19.4)	44 (24.4)	

(brain/lung/bone/liver)										
Radiation	No/unknown	515 (98.5)	177 (98.3)	1	687.6 (98.5)	701.9 (98.6)	0.927	177 (98.3)	177 (98.3)	1
	Yes	8 (1.5)	3 (1.7)		10.6 (1.5)	10.0 (1.4)		3 (1.7)	3 (1.7)	
